# Understanding the Role of Exercise in Nonalcoholic Fatty Liver Disease: ERS-Linked Molecular Pathways

**DOI:** 10.1155/2020/6412916

**Published:** 2020-07-25

**Authors:** Yong Zou, Zhengtang Qi

**Affiliations:** ^1^The Key Laboratory of Adolescent Health Assessment and Exercise Intervention (Ministry of Education), East China Normal University, Shanghai 200241, China; ^2^School of Physical Education and Health, East China Normal University, Shanghai 200241, China

## Abstract

Nonalcoholic fatty liver disease (NAFLD) is globally prevalent and characterized by abnormal lipid accumulation in the liver, frequently accompanied by insulin resistance (IR), enhanced hepatic inflammation, and apoptosis. Recent studies showed that endoplasmic reticulum stress (ERS) at the subcellular level underlies these featured pathologies in the development of NAFLD. As an effective treatment, exercise significantly reduces hepatic lipid accumulation and thus alleviates NAFLD. Confusingly, these benefits of exercise are associated with increased or decreased ERS in the liver. Further, the interaction between diet, medication, exercise types, and intensity in ERS regulation is more confusing, though most studies have confirmed the benefits of exercise. In this review, we focus on understanding the role of exercise-modulated ERS in NAFLD and ERS-linked molecular pathways. Moderate ERS is an essential signaling for hepatic lipid homeostasis. Higher ERS may lead to increased inflammation and apoptosis in the liver, while lower ERS may lead to the accumulation of misfolded proteins. Therefore, exercise acts like an igniter or extinguisher to keep ERS at an appropriate level by turning it up or down, which depends on diet, medications, exercise intensity, etc. Exercise not only enhances hepatic tolerance to ERS but also prevents the malignant development of steatosis due to excessive ERS.

## 1. Introduction

Nonalcoholic fatty liver disease (NAFLD) is characterized by abnormal fat metabolism in the liver due to nonalcoholic causes [1]. NAFLD undergoes a spectrum of pathologies ranging from simple steatosis earlier to nonalcoholic steatohepatitis (NASH) and hepatic fibrosis at the later period [2]. Obesity-related insulin resistance (IR) and ectopic lipid accumulation cause hepatic steatosis [1], followed by increased lipid toxicity, leading to malignant development of liver inflammation and hepatocyte apoptosis [3]. The “two-hits” hypothesis has been the leading theory on NAFLD [4]. The first hit is the excessive accumulation of fat in hepatocytes, leading to IR. The second hit is the inflammatory response in the liver induced by reactive oxygen species (ROS) following the first hit. Recently, endoplasmic reticulum stress (ERS) has been proposed to understand the pathology of NAFLD [5], in which ERS causes hepatic IR, lipid accumulation, inflammation, and hepatocyte apoptosis. Exercise alleviates NAFLD by reducing hepatic lipid and enhancing insulin sensitivity [6–8]. Exercise may modulate ERS levels in multiple organs, leading to improved lipid homeostasis in the liver and even the whole body [9, 10]. Although there is persuasive evidence that exercise reduces hepatic lipid accumulation in humans and animals, ERS may increase or decrease with exercise in these previous studies. If so, what role does ERS play in hepatic lipid accumulation? In this review, we will focus on understanding the role of up- or downregulation of ERS during exercise in NAFLD.

## 2. ERS and Unfolded Protein Response

**Figure 1:**
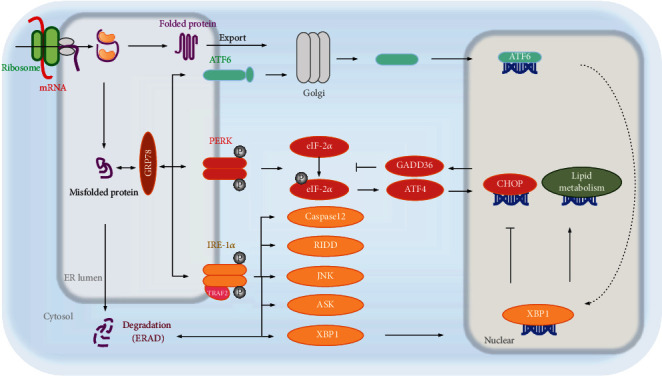
ERS and unfolded protein response (UPR). ATF6, IRE-1*α*, and PERK are the three transmembrane proteins that regulate UPR. GRP78 binds to the three sensor proteins under normal state of protein folding, resulting in UPR silencing. As the misfolded proteins accumulate, GRP78 dissociates from them and binds to the misfolded proteins. Being separated from GRP78, three proteins (ATF6, IRE-1*α*, and PERK) activate the downstream signaling pathway. The activated IRE-1*α* slices the mRNA of XBP1 to form the active transcription factor XBP1s [19]. XBP1s inhibits the expression of CHOP and activates ERAD-mediated unfolded protein degradation [15]. However, chronic ERS initiates the death pathway of IRE-1*α*, including RIDD, to degrade some intracellular mRNA [20]. Meanwhile, IRE-1*α* not only interacts with TRAF2 to activate ASK1 and Caspase12 but also triggers JNK-mediated apoptosis [21]. Activated PERK can phosphorylate eIF-2*α* to halt mRNA translation, leading to the reduction of the initiation complex for the temporary loss of protein synthesis. Phosphorylated eIF-2*α* triggers ATF4 translation, increases the expression of CHOP, and mediates apoptosis [14]. However, chronic ERS induces GADD34 expression to dephosphorylate p-eIF-2*α* [22]. Under ERS, ATF6 translocates from the ER to the Golgi after being dissociated from GRP78 and obtains transcriptional activity through being cleaved by the proteases S1P and S2P. Sliced ATF6 upregulates the expression of XBP1, which results in relieving ERS by enhancing ERAD or inhibiting the expression of CHOP [23]. Together, downstream effects after these signaling pathways include ERAD of misfolded proteins, inhibition of translation, induction of apoptosis or inflammation, and regulation of lipid metabolism. XBP1: X-box binding protein-1; CHOP: C/EBP homologous protein; ERAD: ER-associated degradation; RIDD: regulated IRE-1*α*-dependent decay; TRAF2: TNF-receptor associated factor 2; ASK1: apoptosis signal-regulating kinase; JNK: Jun N-terminal kinase; eIF-2*α*: eukaryotic translation initiation factor 2*α*; ATF4: activating transcription factor 4; GADD34: DNA damage-inducible gene 34.

The endoplasmic reticulum (ER) is a cellular organelle required for calcium homeostasis, protein synthesis, and posttranslational modification and trafficking. ER homeostasis is usually disturbed by numerous environmental, physiological, and pathological insults, referred to as ERS, in which misfolded or incomplete folded proteins are accumulated in the ER, termed the unfolded protein response (UPR) [11–13]. ERS can be provoked by genetic and environmental factors [14, 15]. Cell survival or apoptosis is determined by the UPR during ERS. The UPR is regulated by three transmembrane proteins: protein kinase R-like endoplasmic reticulum kinase (PERK), inositol-requiring protein 1*α* (IRE-1*α*), and activating transcription factor 6 (ATF6). All of them are activated by dissociation with glucose regulated protein 78 kD (GRP78) [16]. GRP78 binds to PERK, IRE-1*α*, and ATF6 in a normal state and dissociates from them when proteins are not fully folded or misfolded. These three protein sensors at ER activate ERS-linked pathways through phosphorylation or translocation, determining cell survival or death under ERS (Figure 1).

Together, the UPR serves as a complex signaling network that restores ER proteostasis and cellular function by increasing protein degradation and reducing protein synthesis to ensure cell survival in transient ERS. However, chronic ERS induces cell apoptosis. ERS often occurs in the liver when hepatocytes are threatened by lipid metabolism disorders, stress, viruses, and other threats. A long-term high-fat diet (HFD) increases the expression of ERS markers in the liver [17]. Persistent hepatic ERS leads to hepatic steatosis, inflammation, and hepatocyte apoptosis, involved in the development of NAFLD [18].

## 3. Exercise and Hepatic Steatosis in Patients with NAFLD

### 3.1. Exercise Types and Diversity

So far, there are no consensual suggestions regarding exercise type to reduce hepatic steatosis. It can be almost inferred from previous studies that each exercise type contributes to liver function, hence avoiding the malignant development of NAFLD. Houghton et al. compared aerobic exercise with resistance exercise for 12 weeks in NAFLD patients, showing that both types of exercise consistently reduced triglyceride (TG) content in the liver and blood glucose [24]. Additionally, 24 weeks of moderate-intensity aerobic exercise improved liver function, as evidenced by serum alanine aminotransferase (ALT) and aspartate aminotransferase (AST) in NAFLD patients [25]. Likewise, Bacchi et al. suggested that both aerobic exercise such as treadmill exercise and resistance training had a similar effect on hepatic TG content in NAFLD patients [26]. These studies suggest that a wide variety of types of exercise contribute to the reduction of NAFLD.

### 3.2. Exercise Intensity

Regarding exercise intensity to reduce hepatic steatosis, a study showed that both high-intensity exercises at 60-80% heart rate (HR) and moderate intensity at 45-55% HR reduced hepatic TG content in NAFLD patients [27]. However, vigorous aerobic exercise at 80% of maximal oxygen consumption was deemed to aggravate NAFLD, which might increase lipid accumulation in the liver due to enhanced lipolysis in peripheral tissues after exercise [28]. Another study showed that high-intensity interval training (HIIT) effectively reduced hepatic TG content in NAFLD patients [29], proving that the benefits of exercise are not limited to exercise intensity. Even though the effects of each exercise intensity are analogous in previous studies, the optimal exercise intensity for personalized NAFLD is still confusing [30, 31].

### 3.3. Exercise Frequency and Duration

The lipid utilization and its beneficial effects may vary with frequency and duration of exercise. However, the frequency and duration of exercise did not limit the risk reduction and development of NAFLD in the follow-up study of NAFLD or non-NAFLD patients. Indeed, the risk reduction and benefit are positively correlated with exercise frequency [32]. Timing exercise bouts to coordinate with individual circadian rhythms might be an effective strategy to optimize the health benefits of exercise [33]. However, an intriguing study showed that the reduction of hepatic TG content did not change with exercise timing [31]. In all, there are still many inconsistencies in the effect of exercise frequency or rhythm on NAFLD. However, exercise with different types, intensity, frequency, and timing has potential to reduce hepatic TG in NAFLD patients, thereby alleviating hepatic steatosis.

## 4. Overview of the Mechanisms by which Exercise Improves Hepatic Steatosis

A large number of studies suggest that exercise improves NAFLD due to reduced hepatic TG and IR. It can be also attributed to the fact that exercise induces weight loss or normal weight [24, 34]. However, amounts of studies have shown that decreased hepatic TG and serum AST and ALT were not related to body weight or body mass index, suggesting that exercise-induced weight control may not play a critical role in NAFLD [30, 35]. Despite the positive effects of exercise on NAFLD, exercise-treated mice showed more severe hepatic steatosis and metabolic disorders compared with mice without exercise, when given a HFD at the same time after exercise training [36]. These contradictions from human and animal experiments prompted us to further explore the molecular mechanisms on NAFLD. To date, studies regarding underlying mechanisms included improving IR, reducing lipid accumulation, suppressing inflammatory pathways, and strengthening cell function to resist the stress. These results were summarized in Table 1. Although some of these results did not mention ERS, we found that the key signaling molecules of ERS were included in effector molecules of exercise. ERS at the subcellular level must be an important mediator for exercise to improve IR, reduce lipid accumulation, relieve liver inflammation, and strengthen cell survival.

## 5. The Up- and Downregulation of ERS in the Liver by Exercise

### 5.1. GRP78 Expression Depends on the Diversity of Exercise Types

GRP78 is the master protein of the UPR and mediates cellular response to normal or stress conditions. GRP78 was significantly reduced in the liver of elderly NAFLD mice after knee loading exercise for 6 weeks, suggesting that ERS was suppressed by resistance exercise [54]. In contrast, Deldicque et al. showed that endurance treadmill exercise for 6 km/week was not a sufficiently strong stimulus to alter the protein expression of GRP78 in elderly obese mice [17]. Similarly, Kristensen et al. demonstrated that swimming exercise was not able to suppress the increased GRP78 in the liver of HFD mice [55]. Astonishingly, results in the referred studies show that lipid accumulation in the liver is all reduced after exercise. As per previous reports, GRP78 expression in the liver depends on the intensity of treadmill exercise [56], where GRP78 functions as a stress-response protein that needs a threshold intensity of exercise stimulation. In this regard, the up- and downregulation of ERS may be related to different types, intensity, and duration of exercise.

### 5.2. The Expression of IRE-1*α*, PERK, and ATF6 Depends on Exercise Conditions

Even though these three signal transducers are activated during ERS, their expression varies greatly under the regulation of exercise. For instance, continuous exhausted exercise reduced ATF6 expression in the liver, while phosphorylation of PERK and IRE-1*α* was not changed [57]. In contrast, another study showed that long-term aerobic exercise suppressed the expression of IRE-1*α* and PERK, rather than ATF6 [55]. In addition, diet also affects the regulation of exercise on ERS in the liver. With the same exercise training, HFD-fed mice showed an increased expression of PERK with unaltered IRE-1*α* and ATF6, while standard diet-fed mice remained unchanged in all these three indicators [58]. Similar results were demonstrated in elderly obese mice [17]. However, 8 weeks of swimming exercise decreased the phosphorylation of PERK in the liver of HFD mice [59]. Several studies with NAFLD rats suggested that swimming exercise, HFD [60], or their combination decreased the expressions of PERK and IRE-1*α* in the liver [61]. The coactivation of ATF6 and PGC-1*α* is related to the exercise adaptability of the skeletal muscle [10]. However, there is no effect of exercise on ATF6 in the liver of elderly mice [55]. During the UPR, the ER transmembrane sensor, including IRE-1*α*, PERK, and ATF6, is activated, by which stress signals are transduced to the outside of the ER, leading to various cell responses including gene induction. In these previous studies, the three sensors had inconsistent responses to exercise, suggesting that the UPR and ERS activation depends on specific exercise and diet conditions (thresholds).

### 5.3. The Regulation of ERS by Exercise Is Affected by Drugs

Despite the increased knowledge of underlying mechanisms on NAFLD, the design of a standard therapeutic approach for such a complex and multifactorial disease seems to be out of reach so far. Exercise combined with drugs as an unconventional therapy intrigues many scholars. Rutin, a glycoside of quercetin, is a nonnutritive component of many foods such as onions, apples, tea, and red wine [62]. As one of the flavonoids, rutin effectively reversed oxidative stress and inflammation in the liver and prevented chronic progression in metabolic syndrome [63]. Exercise and rutin independently did not change the expression of IRE-1*α* in the liver of HFD mice, while their combination significantly reduced the expression of IRE-1*α* [51]. In fact, medication and exercise promote each other to alleviate NAFLD. For instance, an adequate supplement of vitamin D is necessary to achieve the beneficial effects of physical activity [64]. Ezetimibe is useful for treating residual dyslipidemia after exercise intervention in patients with NAFLD [65]. Therefore, it is logically speculated that the combination of exercise and rutin is more useful for NAFLD.

Similar to rutin, resveratrol is a polyphenolic compound, which was regarded as an alternative treatment for NAFLD [66]. Although resveratrol alone has antioxidant, antiapoptotic, and anti-inflammatory properties, combined therapy with interval and continuous training can be more effective to mitigate these abnormalities in NAFLD patients [67]. Exercise combined with resveratrol regulated ERS by different pathways that depend on exercise intensity in a NAFLD model induced by a HFD [68]. However, no evidence clarified why exercise combined with resveratrol suppressed different branches of the UPR at different exercise intensities. Also, we have screened out the potential NAFLD drugs, which combine with exercise to produce a boost by regulating ERS in the liver (Table 2). The pharmacological roles of these drugs fall into four broad categories: cytoprotection (e.g., rutin, resveratrol, vitamin D, vitamin E, betaine, pentoxifylline, UDCA, and silymarin), regulation of lipid metabolism (e.g., statins, ezetimibe, metformin, and omega-3 fatty acids), hormonal regulation (e.g., incretin analogues, TZDs, sitagliptin, and vildagliptin), and interactions of systemic metabolism (e.g., angiotensin receptor blockers, probiotics and synbiotics, and orlistat). Meanwhile, it is necessary to identify whether drugs reduce ERS as a result or cause of the improvement of metabolic disorder. In fact, most of these drugs suppress ERS, and exercise that either increases or decreases ERS seems to have beneficial effects on medication. It is interesting to explore the mechanism underlying the combination of drugs and exercise, supposing combination of both may alleviate hepatic ERS in NAFLD.

Together, ERS in response to exercise is associated with exercise types and intensity, diet, drugs, and other factors. The ERS markers may be either increased or decreased after exercise, but the beneficial effect of exercise on NAFLD is almost consistent in previous studies. Exercise always leads to a positive impact on NAFLD by reducing hepatic lipid content. If ERS is induced by unfolded or misfolded proteins, it may be triggered by the accumulation of misfolded proteins to a certain threshold. Aerobic exercise can prevent accumulated misfolded proteins and reduce oxidative damage, heat-shock protein levels, and exercise tolerance [69]. Exercise-induced metabolic stress could activate the UPR, mediating exercise-induced adaptation responses. In fact, moderate-intensity exercise-induced ERS acts as a protective mechanism against current and future stressors. However, biological responses vary according to exercise intensity and therefore induce different degrees of ERS [70]. Thus, three pathways of ERS may be selectively inhibited by different exercise intensities. These results suggest that the regulation of ERS by exercise may ultimately depend on the extent of misfolded protein accumulation in ER.

## 6. ERS-Related Molecular Mechanism by which Exercise Alleviates NAFLD

### 6.1. Exercise Reduces Lipid Accumulation in the Liver by Regulating ERS

Lipid ectopic accumulation occurs in the early stage of NAFLD, which leads to ERS in the liver [107]. Unfortunately, ERS further promotes lipid overstore and hepatic steatosis and thus leads to NAFLD [14, 108]. Sliced XBP1s is crucial in hepatic lipogenesis. XBP1 enhanced the protein levels of lipogenesis and resulted in lipid accumulation in the liver [109]. XBP1 knockout mice exhibited hypocholesterolemia, hypotriglyceridemia, and reduced liver lipogenesis [110]. Exercise controls the transcription of XBP1 in the liver. The 6-week wheel running suppressed the increase in XBP1s mRNA in HFD-fed mice [17], and similar results were shown in rats after the 6-week treadmill exercise [58]. Furthermore, swimming exercise decreased the protein levels of IRE-1*α* and XBP1 and reduced hepatic TG content in rats with NAFLD [60, 61]. However, exercise also offset the age-induced XBP1s reduction, but not the increased hepatic TG content [55]. Contrary to these previous studies, XBP1 has been identified as an antiadipogenic protein in the liver, which reduces hepatic lipogenic gene expression and improves hepatosteatosis in mouse models of obesity and IR [111].

The biosynthesis of fatty acids and cholesterol is controlled by SREBPs. SREBP is regulated by the PERK pathway in the UPR [112, 113]. Eukaryotic initiation factor eIF-2*α* is phosphorylated by PERK and thus activates SREBP-1c to aggravate hepatic steatosis [114]. ERS induces chronic overexpression of GADD34 [113], dephosphorylates p-eIF-2*α* to inhibit lipid accumulation in the liver. Lipid accumulation and the expression of SREBP-1c were both reduced in the liver of ATF4 knockout mice [115]. Additionally, ATF4 knockout mice showed reduced lipid accumulation in the liver after HFD [116]. Exercise reduced ATF4 protein and TG content in the liver of NAFLD mice [54], indicating that exercise reduced the lipid accumulation by controlling ATF4 expression. Adenosine 5′-monophosphate- (AMP-) activated protein kinase (AMPK) was activated by exercise and suppressed SREBP-1c, thereby reducing lipid accumulation in the liver of HFD mice [41]. Li et al. showed that exercise reduced SREBP-1-induced lipid accumulation in the liver through the AMPK pathway to inhibit the mammalian target of rapamycin complex 1 and relieve ERS [117]. Collectively, exercise reduced hepatic lipogenesis by the PERK/ATF4/SREBP pathway. These studies suggest that exercise regulates hepatic XBP1 and SREBPs through ERS signaling, thereby reducing lipid accumulation in the liver of NAFLD.

In addition, exercise may regulate ERS in adipose tissue to treat NAFLD. ERS promoted lipolysis in adipose tissue and increased circulating free fatty acid (FFA), and FFA is likely to be transferred to the liver for lipid synthesis [118, 119]. Thus, reducing ERS and FFA output from adipose tissue maybe prevents NAFLD. GRP78, PERK, and ATF6 in the subcutaneous adipose tissue of obese individuals were higher than normal, suggesting a higher level of adipocyte ERS with obesity. These ERS markers were decreased by three months of moderate-intensity aerobic exercise, so Khadir et al. conclude that physical exercise alleviates ERS in obese humans through attenuation of the GRP78 signaling network [120]. The regulation of ERS in the skeletal muscle during exercise aroused several scholars' great interest. Kim et al. emphasized the link between ERS in the skeletal muscle and exercise and suggested that ERS was activated in the human skeletal muscle after exercise [121]. Myokines, such as fibroblast growth factor 21 and interleukin-6 (IL-6), were upregulated by enhanced ERS in the skeletal muscle during a short-term exercise [122, 123]. Circulating myokines enhanced *β*-oxidation and repressed lipogenesis in the liver, increased glucose and lipid uptake in adipocytes, and promoted hepatic glycolysis and lipolysis to fuel the muscle [124, 125]. ERS-activated ATF6 upregulated G protein-coupled bile acid receptor 1 expression and increased energy expenditure in the skeletal muscle during exercise [126].

Together, ERS is the trigger of cellular signaling in multiple organs. In the liver, exercise inhibits lipogenesis directly through UPR-mediated cell signaling. In the fat, exercise suppresses ERS to reduce FFA release into the blood, thus reducing fatty acid transport to the liver. In the skeletal muscle, exercise enhances ERS to promote myokine secretion, thereby targeting the liver to promote lipolysis. Anyway, exercise can reduce lipid accumulation in the liver through the modulation of ERS.

### 6.2. Exercise Reduces Insulin Resistance in the Liver by Regulating ERS

IR is closely associated with NAFLD, while it is hard to clarify which is the cause and which is the result. ERS is essential for FFA-induced inflammation and IR with PERK and I*κ*B kinase-*β* (IKK*β*) as the critical signaling components. Deficiency of PERK attenuates FFA-induced activation of IKK*β*, and deficiency of IKK*β* alleviates FFA-induced inflammation and IR [127]. Also, PERK acts oppositely to promote Forkhead box protein O (FOXO) activity via phosphorylation of FOXO1 and results into IR. Inhibition of PERK improves cellular insulin responsiveness at the level of FOXO activity [128]. By the IRE-1*α* pathway, XBP1s interacts with FOXO1 and thus bypasses hepatic IR independent of its effects on ER protein folding. Modest hepatic overexpression of XBP1s in mouse models of insulin deficiency or IR reduced blood glucose, without improving insulin signaling and ER folding capacity [129]. Activated IRE-1*α* leads to suppression of insulin receptor signaling through hyperactivation of JNK and subsequent serine phosphorylation of insulin receptor substrate-1 (IRS-1). The IRE-1*α*-JNK signaling pathway can directly inhibit cytoplasmic insulin signaling because activated JNK phosphorylates IRS-1 at Ser307 [130]. Thus, ERS impairs insulin signaling and results into IR.

Exercise improves IR in the liver by reducing ERS. For instance, swimming exercise led to reduction in PERK and eIF-2*α* phosphorylation and reduced proinflammatory molecules (JNK, I*κ*B, and NF-*κ*B) in the liver, with enhanced insulin signaling [59]. However, Deldicque et al. found that phosphorylation of JNK and IKK was increased in the liver with unimproved glucose tolerance, although 6-week endurance exercise reduced the phosphorylation of IKK in the obese mice. The authors conclude that the potentiation of the UPR by endurance training may represent a positive adaptation protecting against further cellular stress [17]. These opposite results still show that JNK is linked to the insulin signaling, whether exercise reduces or increases ERS. In line with PERK, the phosphorylation of IRE-1*α* and JNK decreased in the liver of HFD mice after 16 weeks of treadmill exercise [51]. Thus, an appropriate exercise may improve insulin sensitivity through the PERK/JNK or IRE-1*α*/JNK pathways to reduce hepatic IR.

Unlike acute exercise, several training sessions weakened ERS in the skeletal muscle. Aging induced oxidative stress and activated ERS-related apoptosis in the skeletal muscle, whereas long-term wheel-running improved redox regulation, ERS adaptation, and attenuated ERS-related apoptosis [131]. Accumulating studies demonstrated that ERS negatively regulated skeletal muscle insulin sensitivity. Overproduction of ATF6 is sufficient to inhibit the expression of glucose transporter 4 in the skeletal muscle [132]. Also, the upregulated Tribbles 3 by ERS, as a pseudokinase in the skeletal muscle [133], inhibited phosphorylation of Akt and repressed glucose uptake [134]. Hepassocin (HPS) is a novel hepatokine that causes hepatic steatosis. ERS induced by palmitate could increase the expression of HPS in hepatocytes and further contribute to the IR in the skeletal muscle via the epidermal growth factor receptor (EGFR)/JNK-mediated pathway [135]. In turn, ERS induced by exercise in the skeletal muscle could increase the release of myokines such as FGF21 and IL-6 and further reduce hepatic IR in NAFLD ultimately [122, 123].

### 6.3. Exercise Modulates the Inflammatory Response in the Liver by Regulating ERS

Currently, IR and mild lipid accumulation in the liver are considered common symptoms of reversible NAFLD. However, the UPR cannot restore ER homeostasis in the later stage of NAFLD, and chronic inflammation promotes the further deterioration of fatty liver [136]. ROS-induced lipid peroxidation is more and more serious and triggers inflammation in the liver [137]. In fact, the hepatic inflammation and the UPR occur simultaneously by the IRE-1*α* and PERK pathways. IRE-1*α* triggers the JNK inflammatory pathway, thus phosphorylating I*κ*B and activating NF-*κ*B [138]. PERK is activated by ERS, and the resulting eIF-2*α* phosphorylation is critical for the activation of NF-*κ*B [139], where the decreased I*κ*B/NF-*κ*B ratio promotes inflammation during ERS [140]. Furthermore, increased thioredoxin interaction protein (TXNIP) induced by IRE-1*α* and PERK, which interacted with ROS, thus aggravated the inflammatory response [141]. In addition, ERS elevated hepatic sensitivity to lipotoxicity and release of inflammatory cytokines to activate more macrophages [142].

For liver inflammation, studies regarding the regulation of exercise on ERS focus on the PERK and IRE-1*α* pathways. Lifelong exercise significantly reduced the phosphorylation of IRE-1*α* and JNK in the liver [55], suggesting that exercise might alleviate the inflammation through regulating the IRE-1*α* pathway. Moreover, endurance exercise decreased JNK, I*κ*B, and NF-*κ*B levels in the liver of obese mice, with a significant reduction of PERK and eIF-2*α* [59]. In contrast to these results, Deldicque et al. showed that aerobic exercise enhanced ERS and the phosphorylation of JNK, with higher levels of IL-1 and IL-6 in the liver [17]. Interestingly, hepatic metabolic abnormality in these mice was all improved, suggesting that reducing ERS and inflammation may not be necessary for exercise to alleviate NAFLD.

Together, increasing and decreasing ERS with exercise both leads to health benefits. By this way, ERS results in enhancement or reduction of inflammation. For exercise, studies have demonstrated the effects of aerobic exercise on ERS-induced inflammation, while the effects of resistance exercise have not been well-established. Endurance exercise either reduces hepatic ERS as a pathway for reducing inflammation in liver or increases hepatic ERS and inflammation as a stimulus to elevate the anti-inflammatory threshold protecting against further cellular stress. In any case, cells are likely to be protected by exercise. Previous conclusions about exercise effects are contradictory, which may be related to the development stage of NAFLD and exercise conditions. It is worth discussing that the type and intensity of exercise must be highly compatible with the stage of NAFLD or it may backfire.

### 6.4. Exercise Controls Hepatic Lipoapoptosis by Regulating ERS

A large number of hepatocytes undergo apoptosis and are replaced by fibrosis, leading to severe deterioration in the later stage of NAFLD. Hepatocyte apoptosis with lipotoxicity, referred to as lipoapoptosis, is mainly induced by ERS and the mitochondrial apoptotic pathway in NAFLD [143]. Saturated FFA induces JNK-dependent hepatocyte lipoapoptosis by activating the proapoptotic factor, which triggers the mitochondrial apoptotic pathway. Although saturated and monounsaturated FFAs caused equal cellular steatosis, apoptosis and JNK activation were greater during exposure to saturated versus monounsaturated FFAs [144]. In human and mouse hepatocytes, palmitic acid at a lipotoxic concentration triggered early activation of ERS-related kinases, induced the ERS related-apoptotic transcription factor CHOP, activated Caspase3, and increased the percentage of apoptotic cells [145]. There are three types of lipoapoptosis, which are mediated by UPR, resulted from calcium (Ca^2+^) disorder in the ER lumen, and induced by an ERS-specific apoptotic protein Caspase12 [143, 146, 147]. Moreover, ERS-mediated lipoapoptosis in the liver is induced by the PERK and IRE-1*α* pathways.

Exercise reduces hepatocyte lipoapoptosis in mice with NAFLD. Resveratrol, exercise, and their combination reduced the number of apoptotic cells significantly in the liver [148]. Furthermore, moderate-intensity aerobic exercise combined with resveratrol reduced IRE-1*α* and PERK levels, with their downstream proteins such as JNK1 and CHOP, as well as Caspase3 and Bcl-2-associated X protein (BAX), the apoptosis proteins [68]. This indicates that exercise combined with resveratrol may reduce the hepatocyte apoptosis through the IRE-1*α*/JNK and PERK/CHOP pathways in mice with NAFLD. 8 weeks of exhausting exercise decreased the expression of Caspase3 and ATF6 in the liver [57]. Consecutive aerobic exercise for 8 weeks with HFD cancellation suppressed CHOP, Caspase12, and JNK, with decreased apoptotic hepatocytes in mice with NAFLD [149]. Thus, exercise reduces lipoapoptosis through the IRE-1*α*/JNK and PERK/CHOP apoptotic pathways.

However, Deldicque et al. showed that endurance exercise increased PERK and IRE-1*α* and enhanced Caspase12 in the liver of obese mice [17]. The antiapoptotic factor Bcl-2 was inactivated by the JNK pathway, which may mediate the increasing hepatocyte apoptosis during endurance exercise. Double-KO (proapoptotic Bcl-2 family members BAX and BAK) mice responded abnormally to tunicamycin-induced ERS in the liver, with extensive tissue damage and decreased expression of the IRE1 substrate XBP1 and its target genes [150]. Another study also showed that low-intensity aerobic exercise could not reduce hepatic lipoapoptosis in mice with NAFLD [68]. As previously mentioned, the effect of exercise on ERS is inconsistent in separate studies. Nevertheless, what is relatively clear is that exercise reduces hepatocyte apoptosis by relieving ERS in NAFLD.

In addition, exercise significantly reduced the expression of Caspase12 in the liver of NAFLD [151], indicating that exercise might decrease Caspase12 to inhibit hepatocyte apoptosis. Also, aerobic exercise with different intensities reduced the expression of Caspase12 and the hepatocyte lipoapoptosis in diabetic mice [56]. However, Kristensen et al. demonstrated no difference in the Bax/Bcl-2 ratio in the livers of untrained and trained older mice, suggesting that exercise did not reduce hepatocyte apoptosis [55]. Additionally, exercise reduced GRP78, CHOP, and cleaved Caspase12 protein in an intensity-dependent manner. Exercise appeared to ameliorate diabetic cardiomyopathy by inhibiting ERS-induced apoptosis in diabetic rats [152]. Dietary obesity could induce prefrontal ERS in rats, and excessive ERS decreases the levels of neuroplasticity-associated proteins. Exercise could reduce GRP78, p-PERK, p-eIF-2*α*, Caspase12, CHOP, and Bax/Bcl-2 expressions and ERS-induced apoptosis, thus promoting the expression of neuroplasticity-associated proteins [153]. Although these studies were not about NAFLD, the findings suggest that exercise has significant tissue specificity in regulating apoptosis through ERS.

## 7. Conclusion and Remarks

**Figure 2:**
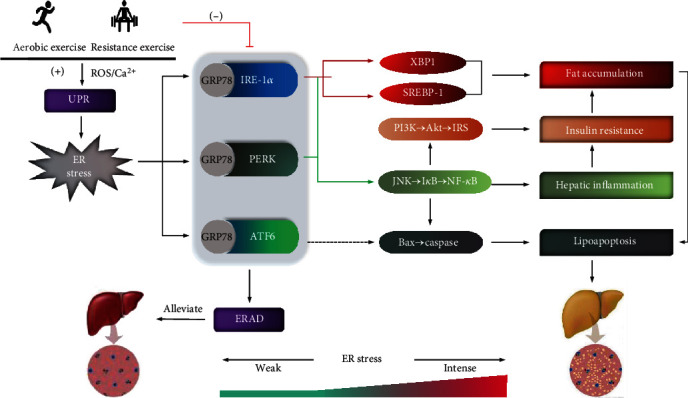
ERS-linked molecular pathways by which exercise alleviates NAFLD. Exercise maintains ERS at a certain level essential for hepatic lipid homeostasis. First, acute exercise including aerobic and resistance exercise induces ROS production and Ca^2+^ disorders, which evoke the UPR and the resulting ERS. This is conducive to elevate hepatic tolerance against higher ERS and the clearance of misfolded proteins. Second, regular exercise suppresses the activation of UPR and excessive ERS, thereby reducing lipid accumulation, inflammation, insulin resistance, and hepatocyte lipoapoptosis through IRE-1*α*- and PERK-mediated pathways.

In conclusion, exercise alleviates NAFLD by reducing lipid accumulation, insulin resistance, hepatocyte lipoapoptosis, and the inflammatory response. These improvements are associated with ERS at the subcellular level in the liver and beyond. However, not all of exercise protocols produce similar ERS. Exercise effects on ERS depend on a variety of conditions, such as the type and intensity of exercise, diet, medications, age, and pathology. Nonetheless, most studies support the benefits of exercise for NAFLD. First, exercise can trigger the UPR to enhance hepatic threshold of ERS tolerance, suggesting that the liver can withstand higher levels of misfolded proteins and raise the protein clearance efficiency. Second, regular exercise can prevent the malignant development of NAFLD by suppressing excessive ERS at the downstream of the UPR. Therefore, ERS must be an intracellular signal of lipid homeostasis for the liver. Too low ERS is not conducive to the clearance of misfolded proteins, whereas too high ERS may activate inflammatory signaling pathways and hepatic apoptosis. Moderate-intensity acute exercise combined with regular exercise produces a moderate level of ERS for hepatic lipid homeostasis (Figure 2). In addition, exercise effects on ERS (up- or downregulation) in the skeletal muscle, fat, and even brain contribute to lipid turnover in the liver through UPR-mediated cytokine secretion. Further studies are needed to explore how the up- and downregulation of ERS in different tissues and organs during exercise are well orchestrated to reduce lipid accumulation in the liver.

## Figures and Tables

**Table 1:** Summary of the effects of exercise on NAFLD and related molecular mechanisms.

Role	Animal	Exercise types	Results and mechanisms	Reference
Reducing lipid accumulation	Mice	Treadmill exercise; 60 min/d, 6 weeks	Lipid droplets reduction, nitric oxide content ↑, total nitric oxide synthase (NOS) ↑, inducible NOS ↑, endothelial NOS, inducible NOS mRNA ↑	[37]
	Rat	Swimming; 90 min/d, 12 weeks	Serum adiponectin (ADPN) ↑, liver adiponectin receptor2 (AdipoR2) ↑, peroxisome proliferator-activated receptor *α* (PPAR*α*) ↑	[38]
	Rat	Swimming; 90 min/d, 12 weeks	p-AMPK ↑, p-AMPK/AMPK ↑	[39]
	Mice	Treadmill exercise; 60 min/d, 7 weeks	Liver thiobarbituric acid reactive substances ↓, glutathione peroxidase, SOD, liver *β*-oxidation ↑, tumor necrosis factor-*α* (TNF-*α*) ↓, PPAR*α* ↑, alternative oxidase, carnitine palmitoyltransferase 1, long-chain acyl-CoA dehydrogenase, cytochrome P450 4A10 (CYP4A10), CYP4A12 mRNA ↑	[40]
	Rat	Swimming; 60-90 min/d, 12 weeks	p-Protein kinase B (p-Akt) ↑, AMPK staining ↑, PPAR staining ↑, sterol regulatory element-binding protein 1c (SREBP-1c), stearoyl-CoA desaturase 1 ↓	[41]
	Rat	Treadmill exercise; 60 min/d, 6 weeks	Mannosylglycoprotein N-acetyl-glucosaminyltransferase 1 ↓, PPAR*γ* ↑, carbohydrate response element binding protein, SREBP-1c ↓	[42]
Improving insulin resistance	Mice	Treadmill exercise (HIIT) 60 min/d, 6 weeks	p-Akt ↑, fat and glucose uptake ↑, 2-deoxy-D-glucose uptake ↑, inflammatory factors remained stable	[43]
	Rat	Swimming; 60 min/d, 6 weeks	Serum Irisin ↑, liver PPAR*α* ↑	[44]
	Human	Brisk walking, tai chi; 60 min/d, 16 weeks	Serum SREBP-1c ↓, retinol binding protein 4 ↓, TNF-*α* ↓	[45]
Inhibiting inflammation	Rat	Treadmill exercise 60 min/d, 6 weeks	High-molecular-weight adiponectin ↑, Sirt-1 ↑, nuclear factor kappa-B (NF-*κ*B) ↓, monocyte chemotactic protein 1, neutrophil cytosolic factor 2 ↓	[46]
	Rat	Swimming; 90 min/d, 12 weeks	NF-*κ*B ↓, TNF-*α* ↓	[47]
	Mice	Treadmill exercise; 60 min/d, 16 weeks	TNF-*α* ↓, tissue inhibitors of metalloproteinase 1 ↓, collagen1*α* ↓, CD11c ↓, toll-like receptor 4 ↓	[48]
	Rat	Swimming; 90 min/d, 12 weeks	TNF-*α* ↓, adiponectin mRNA ↑	[49]
	Rat	Swimming; 90 min/d, 12 weeks	Serum ADPN ↑, liver AdipoR2 ↑, PPAR*α* ↑	[38]
Strengthening cell function	Mice	Treadmill exercise; 40 min/d, 10 weeks	Liver miR33 ↑, fatty acid synthase, acetyl-coa carboxylase, SREBP-1c ↓, liver autophagy-related protein 5, autophagy-related protein 7, lysosomal-associated membrane protein 2, Beclin1 ↑	[50]
	Mice	Treadmill exercise; 60 min/d, 16 weeks	Protein dispersibility index ↓, IRE-1*α*, p-JNK ↓, phosphoenolpyruvate carboxykinase ↓, peroxisome proliferator-activated receptor *γ* coactivator 1*α* (PGC-1*α*) ↑	[51]
	Rat	Wheel running; 60 min/d, 8 weeks	Mitochondrial metabolic enzyme ↑, oxygen consumption ↑, maintain mitochondrial membrane phosphatidylethanolamine	[52]
	Rat	Treadmill exercise; 60 min/d, 8 weeks	Liver mitochondrial phosphatidylcholine, phosphatidylinositol ↑, saturated fatty acids ↓, polyunsaturated fatty acids ↑, mitochondrial biogenesis ↑	[53]

**Table 2:** The potential drugs combined with exercise to regulate ERS in NAFLD.

Drugs	Pharmacological role	Effects on NAFLD	Exercise effects on medication	Drug effects on ERS
Rutin	Cytoprotection and antioxidant	Abdominal fat ↓, glucose tolerance ↑, hepatic function ↑, inflammation ↓, ROS ↓	↑ [51]	↓ [51]
Resveratrol	Cytoprotection	Hepatic apoptosis ↓, liver ERS ↓, hepatic steatosis ↓	↑ [67]	↓ [68]
Vitamin D	Antioxidants	IR ↓, serum lipid level ↓, ALT ↓	↑ [64]	↓ [71]
Vitamin E	Antioxidants	Lipid peroxidation ↓, inflammation ↓, hepatic steatosis ↓, AST ↓, ALT ↓	↑ [72]	↓ [73]
Betaine	Antioxidants and insulin sensitizers	Adipocytokines ↑, IR ↓, adipose ERS ↓	↑ [74]	↓ [75]
Pentoxifylline	Cytoprotection (low ROS and inflammation)	ALT ↓, AST ↓, inflammation ↓	↑ [76]	↓ [77]
UDCA	Cytoprotection	No effective improvement	?	↓ [78, 79]
Silymarin	Cytoprotection and antioxidant	ALT ↓, high-density lipoprotein cholesterol ↑, oxidative stress ↓	?	↓ [80]
Statins	Reduce lipid level	Adipocytokines ↑, ALT, AST ↓, inflammation ↓, serum lipid level ↓	↑ [81]	↓ [82]
Ezetimibe	Reduce lipid level	Low-density lipoprotein cholesterol ↓, serum TG ↓, IR ↓	↑ [65, 83]	↓ [84]
Metformin	AMPK activator (promote fat consumption)	ALT ↓, inflammation ↓, hepatocellular injury ↓	↑ [85]	↓ [86, 87]
Omega-3 fatty acids	Improve metabolic profiles	Hepatic steatosis ↓, insulin sensitivity ↑	↑ [88, 89]	↓ [90, 91]
Incretin analogues	Modulate secretion of hormone	Glycaemic levels ↓, hepatic steatosis ↓, inflammation ↓, glycaemic levels ↓, fibrosis ↓	↑ [92, 93]	↓ [94, 95]
TZDs (pioglitazone)	Insulin sensitizers	ALT, AST↓, hepatic steatosis ↓, TG ↓, adipose IR ↓, inflammation ↓	↑ [96]	↓ [97, 98]
Sitagliptin and vildagliptin	DPP-4 inhibitors (suppress the degradation of glucagon-like peptide 1)	No effective improvement	?	↓ [99, 100]
Angiotensin receptor blockers	Modulate the renin-angiotensin-aldosterone system	ALT ↓, inflammation ↓	↑ [101]	↓ [102]
Probiotics and synbiotics	Improve inflammatory conditions of the gastrointestinal tract	Liver ROS ↓, inflammation ↓, ALT ↓	↑ [103]	↓ [104]
Orlistat	Augment weight loss and inhibit fat absorption	AST/ALT↓, hepatic steatosis ↓, weight ↓, ROS ↓	↑ [105, 106]	?
